# Supplementary material for: Aerosol and surface stability of HCoV-19 (SARS-CoV-2) compared to SARS-CoV-1
Source: medRxiv. 2020 Mar 13:2020.03.09.20033217. Originally published 2020 Mar 13. Preprint. [Version 2] doi: 10.1101/2020.03.09.20033217 (PMC7217062; doi:10.1101/2020.03.09.20033217)
Supplement: Supplement 2020 [file 73540-2020.03.09.20033217-1.docx]

**Supplemental Appendix**

# Aerosol and surface stability of SARS-2 (HCoV-19) compared to SARS-CoV

Neeltje van Doremalen^1*^, Trenton Bushmaker^1*^, Dylan H. Morris^2*^, Myndi G. Holbrook^1^, Amandine Gamble^3^, Brandi N. Williamson^1^, Natalie J. Thornburg^4^, Susan I. Gerber^4^, James O. Lloyd-Smith^3,5^, Emmie de Wit^1^, Vincent J. Munster^1^

1. Laboratory of Virology, Division of Intramural Research, National Institute of Allergy and Infectious Diseases, National Institutes of Health, Hamilton, MT, USA
2. Dept. of Ecology and Evolutionary Biology, Princeton University, Princeton, NJ, USA
3. Dept. of Ecology and Evolutionary Biology, University of California, Los Angeles, Los Angeles, CA, USA
4. Division of Viral Diseases, National Center for Immunization and Respiratory Diseases, Centers for Disease Control and Prevention, Atlanta, GA, USA.
5. Fogarty International Center, National Institutes of Health, Bethesda, MD, USA

^*^ These authors contributed equally to this article

# Supplemental methods

***Laboratory experiments***

Viruses and titration

HCoV-19 nCoV-WA1-2020 (MN985325.1) (Holshue et al., 2020) and SARS-CoV-1 Tor2 (AY274119.3) (Marra et al., 2003) were the strains used in our comparison. Viable virus in all surface and aerosol samples was quantified by end-point titration on Vero E6 cells as described previously (van Doremalen et al., 2013).

Virus stability in aerosols

Virus stability in aerosols was determined as described previously at 65% relative humidity (RH) and 21-23°C (Fischer et al., 2016). In short, aerosols (<5 µm) containing HCoV-19 (10^5.25^ TCID_50_/mL) or SARS-CoV-1 (10^6.75-7^ TCID50/mL) were generated using a 3-jet Collison nebulizer and fed into a Goldberg drum to create an aerosolized environment. Aerosols were maintained in the Goldberg drum and samples were collected at 0, 30, 60, 120 and 180 minutes post-aerosolization on a 47mm gelatin filter (Sartorius). Filters were dissolved in 10 mL of DMEM containing 10% FBS. Three replicate experiments were performed.

Virus stability on surfaces

Surface stability was evaluated on plastic (polypropylene, ePlastics), AISI 304 alloy stainless steel (Metal Remnants), copper (99.9%) (Metal Remnants) and cardboard (local supplier) representing a variety of household and hospital situations and was performed as described previously at 40% RH and 21-23°C using an inoculum of 10^5^ TCID_50_/mL (van Doremalen et al., 2013). This inoculum resulted in cycle-threshold values (Ct) between 20 and 22 similar to those observed in samples from human upper and lower respiratory tract (Zou et al., 2020). In short, 50 µl of virus was deposited on the surface and recovered at predefined time-points by adding 1 mL of DMEM. Stability on cardboard was evaluated by depositing 50 µl of virus on the surface and recovering the inoculum by swabbing of the surface, the swab was deposited 1 mL of DMEM. Three replicate experiments were performed for each surface.

***Statistical analyses***

Bayesian regression model description

The durations of detectability depend on initial inoculum and sampling method, as expected. To evaluate the inherent stability of the viruses, we estimated the decay rates of viable virus titers using a Bayesian regression model. This modeling approach allowed us to account for differences in initial inoculum levels across replicates, as well as interval-censoring of titer data and other sources of experimental noise. The model yields estimates of posterior distributions of viral decay rates and half-lives in the various experimental conditions – that is, estimates of the range of plausible values for these parameters given our data, with an estimate of the overall uncertainty (Gelman et al., 2013).

In the model notation that follows, the symbol ~ denotes that a random variable is distributed according to the given distribution. Normal distributions are parametrized as Normal(mean, standard deviation). Positive-constrained normal distributions (“Half-Normal”) are parametrized as Half-Normal(mode, standard deviation). We use <Distribution Name>CDF(x, parameters) to denote the cumulative distribution function of a probability distribution, so for example NormalCDF(5, 0, 1) is the value of the Normal(0, 1) cumulative distribution function at 5.

Our data consist of 10 experimental conditions: 2 viruses (HCoV-19 and SARS-CoV-1) by 5 environmental conditions (aerosols, plastic, stainless steel copper and cardboard). Each has three replicates, and multiple time-points for each replicate. We analyze the two viruses separately. For each, we denote by y_ijk_ the measured log_10_ titer in experimental condition *i* during replicate *j* at time-point *k*. To construct our likelihood function, we need to know the probability of observing a given log_10_ titer measurement y_ijk_ given values of the parameters.

Because our titer data are estimated and recorded in increments of 1/n_wells_ log_10_TCID_50_/mL, where n_wells_ is the number of wells used for endpoint titration, our log_10_ titer values are interval-censored – only known to within a range of width 1/n_wells_. In addition, there is a degree of measurement noise in the titration process itself.

To model this, we assume that in each experimental condition *i*, there is a true underlying log_10_ titer x_ijk_ that is measured with some amount of experimental noise or error ε_ijk_ and then observed as an interval-censored value y_ijk_ ≈ x_ijk_ + ε_ijk_. We model the measurement errors ε_ijk_ as Normally distributed with a standard deviation σ_i_ that is shared by all samples in the given experimental condition; this reflects the fact that some experimental setups may be more or less noisy than others.

ε_ijk_ ~ Normal(0, σ_i_)

We model the probability of observing an interval-censored log_10_ titer value y_ijk_ given a true underlying log_10_ titer x_ijk_ and a measurement error standard deviation σ_i_ as:

P(y_ijk_ | x_ijk_, σ_i_ ) = NormalCDF(y_ijk_, x_ijk_, σ_i_) – NormalCDF(y_ijk_ – 1/n_wells_, x_ijk_, σ_i_)

This reflects the probability given a true value x_ijk_ plus the measurement error x_ijk_ + ε_ijk_ falls between y_ijk_ – 1/n_wells_ and y_ijk_. Due to the log_10_ titer imputation technique used, a titer in that range is most likely to be rounded up and reported as y_ijk_.

The detection limit of our experiment is 0.5 log_10_ TCID_50_/mL. The probability of observing an undetectable measured log_10_ titer value y_ijk_ given a true log_10_ titer value x_ijk_ is given by:

P(y_ijk_ ≤ 0.5 | x_ijk_, σ_i_) = NormalCDF(0.5, x_ijk_, σ_i_)

We then model each replicate *j* for experimental condition *i* as starting with some true initial log_10_ titer x_ij_(0) = x_ij0_. We assume that viruses in experimental condition *i* decay exponentially at a rate λ_i_ over time *t*. It follows that

x_ij_(t) = x_ij0_ – λ_i_t

where t_k_ is the k^th^ measured time-point.

Model prior distributions

We place a weakly informative Normal prior distribution on the initial log_10_ titers x_ij0_ to rule out implausibly large or small values (e.g. in this case undetectable log_10_ titers or log_10_ titers much higher than the deposited concentration), while allowing the data to determine estimates within plausible ranges:

x_ij0_ ~ Normal(4.5, 2.5)

We likewise placed a weakly informative Half-Normal prior on the exponential decay rates λ_i_:

λ_i_ ~ Half-Normal(0.5, 4)

We placed a weakly informative Half-Normal prior on the standard deviations of the experimental error distributions σ_i_:

σ_i_ ~ Half-Normal(0, 2)

Markov Chain Monte Carlo Methods

We drew posterior samples using Stan, which implements a No-U-Turn Sampler (a form of Markov Chain Monte Carlo). We ran four replicate chains from random initial conditions for 2000 iterations, with the first 1000 iterations as a warmup/adaptation period. We saved the final 1000 iterations from each chain, giving us a total of 4000 posterior samples. We assessed convergence by inspecting trace plots and examining *R̂* and effective sample size (n_eff_) statistics (*R̂* for all parameters ≤ 1.003, n_eff_ for all parameters ≥28% of total samples).

# Supplemental table and figures

Table 1. Posterior median estimates and 95% credible intervals (2.5%–97.5% quantile range) for half-lives of HCoV-19 and SARS-CoV-1 in aerosols and on various surfaces, as well as a median estimate and 95% credible interval for the difference between the two half-lives (HCoV-19 – SARS-CoV-1).

|  | **HCoV-19** | | | | **SARS-CoV-1** | | | **HCoV-19 – SARS-CoV-1** | | |
| --- | --- | --- | --- | --- | --- | --- | --- | --- | --- | --- |
|  | half-life (hrs) | | | | half-life (hrs) | | | difference (hrs) | | |
| *Material* | *median* | *2.5%* | *97.5%* | *median* | | *2.5%* | *97.5%* | *median* | *2.5%* | *97.5%* |
| Aerosols | 1.09 | 0.64 | 2.64 | 1.18 | | 0.778 | 2.43 | -0.0913 | -1.35 | 1.39 |
| Copper | 0.774 | 0.427 | 1.19 | 1.5 | | 0.929 | 2.66 | -0.735 | -1.91 | -0.0339 |
| Cardboard | 3.46 | 2.34 | 5 | 0.587 | | 0.317 | 1.21 | 2.85 | 1.58 | 4.41 |
| Steel | 5.63 | 4.59 | 6.86 | 4.16 | | 3.3 | 5.22 | 1.46 | 0.00127 | 2.96 |
| Plastic | 6.81 | 5.62 | 8.17 | 7.55 | | 6.29 | 9.04 | -0.722 | -2.64 | 1.16 |

Figures S1–S5 (below) show Bayesian fits to individual replicate virus decay data for each virus. Replicates are shown in panel columns, viruses in panel rows. Lines are 50 random draws per panel from the posterior distribution of fitted lines, to show level of uncertainty. Time axis is shown out to the latest time taken to reach an undetectable titer in the considered experimental conditions.


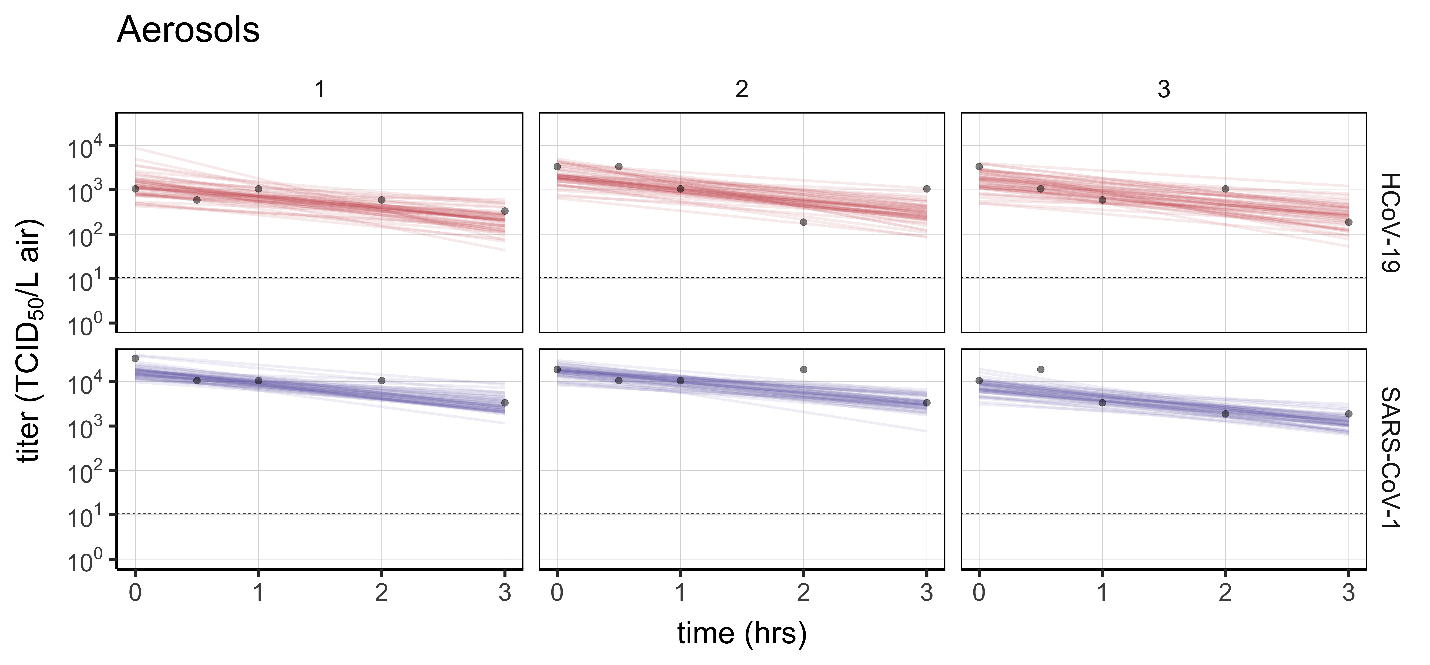


Figure S1. Individual replicate fits for aerosols. Columns show replicates, rows show virus (HCoV-19 above, SARS-CoV-1 below). Lines are 50 random draws per panel from the posterior distribution of fitted lines, to show level of uncertainty.


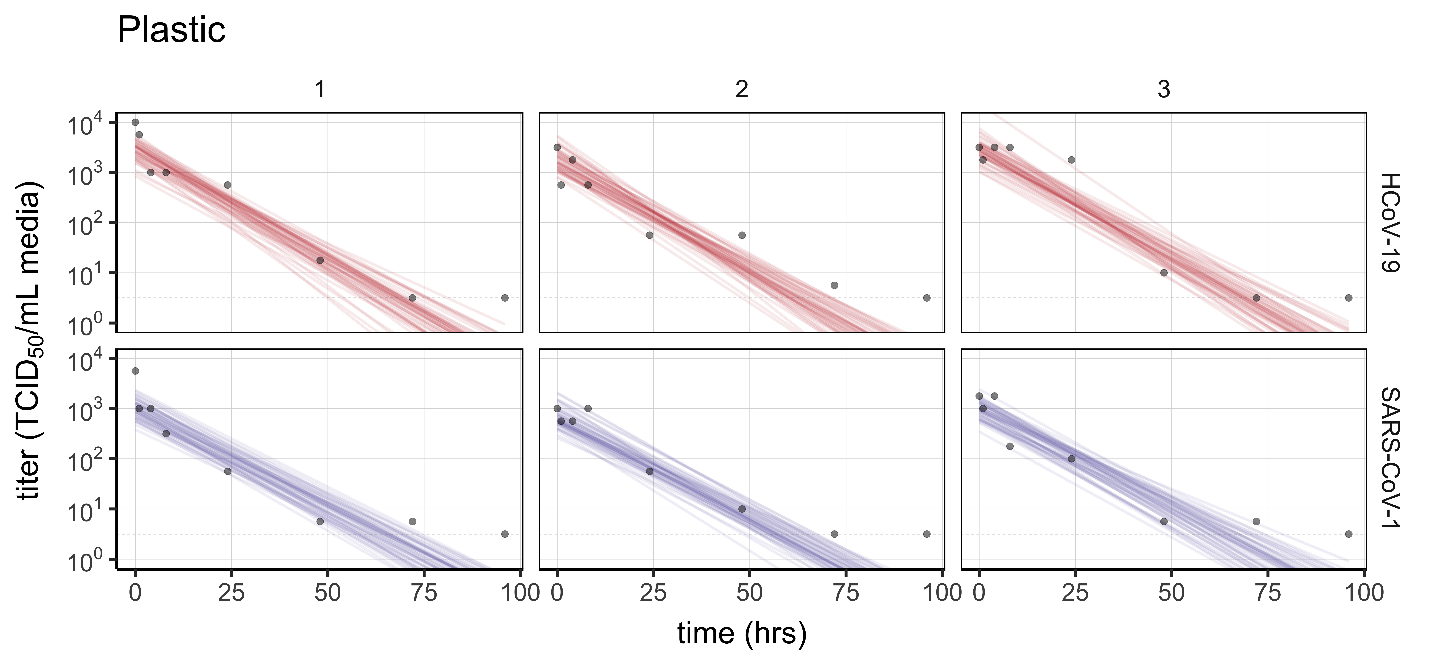


Figure S2. Individual replicate fits for plastic. Columns show replicates, rows show virus (HCoV-19 above, SARS-CoV-1 below). Lines are 50 random draws per panel from the posterior distribution of fitted lines, to show level of uncertainty.


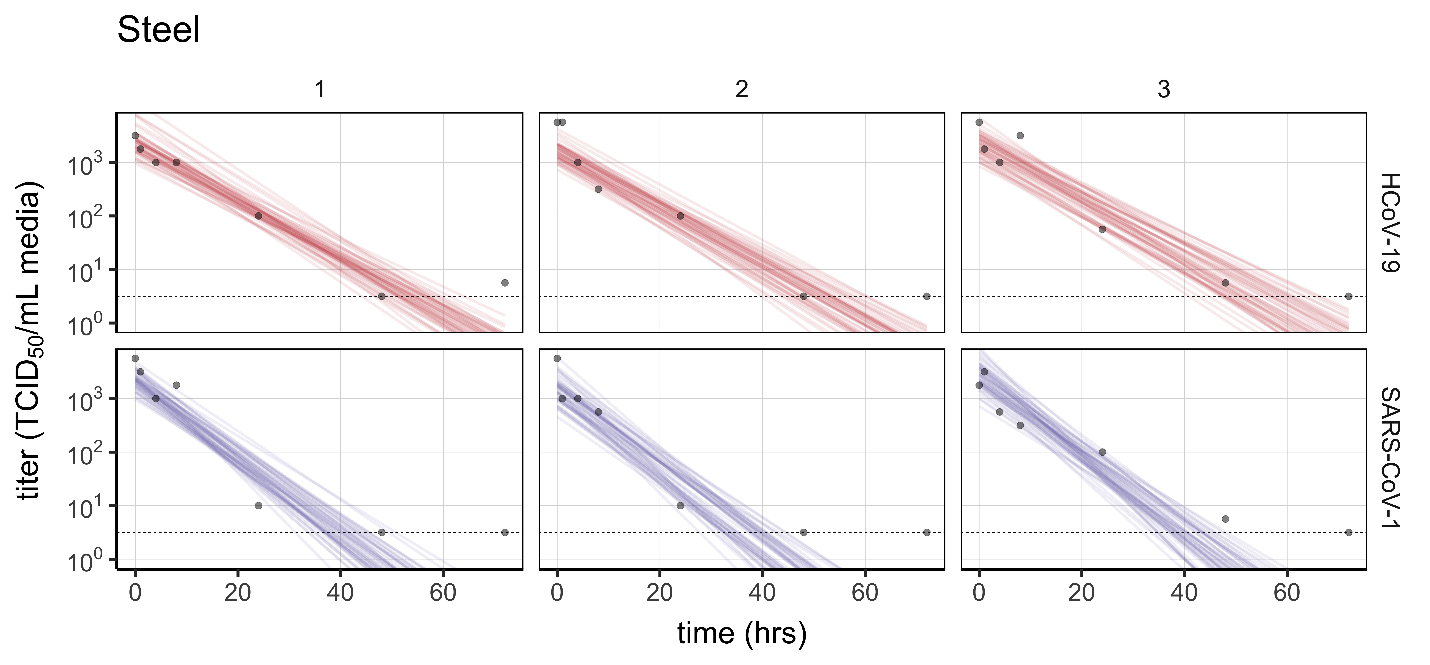


Figure S3. Individual replicate fits for steel. Columns show replicates, rows show virus (HCoV-19 above, SARS-CoV-1 below). Lines are 50 random draws per panel from the posterior distribution of fitted lines, to show level of uncertainty.


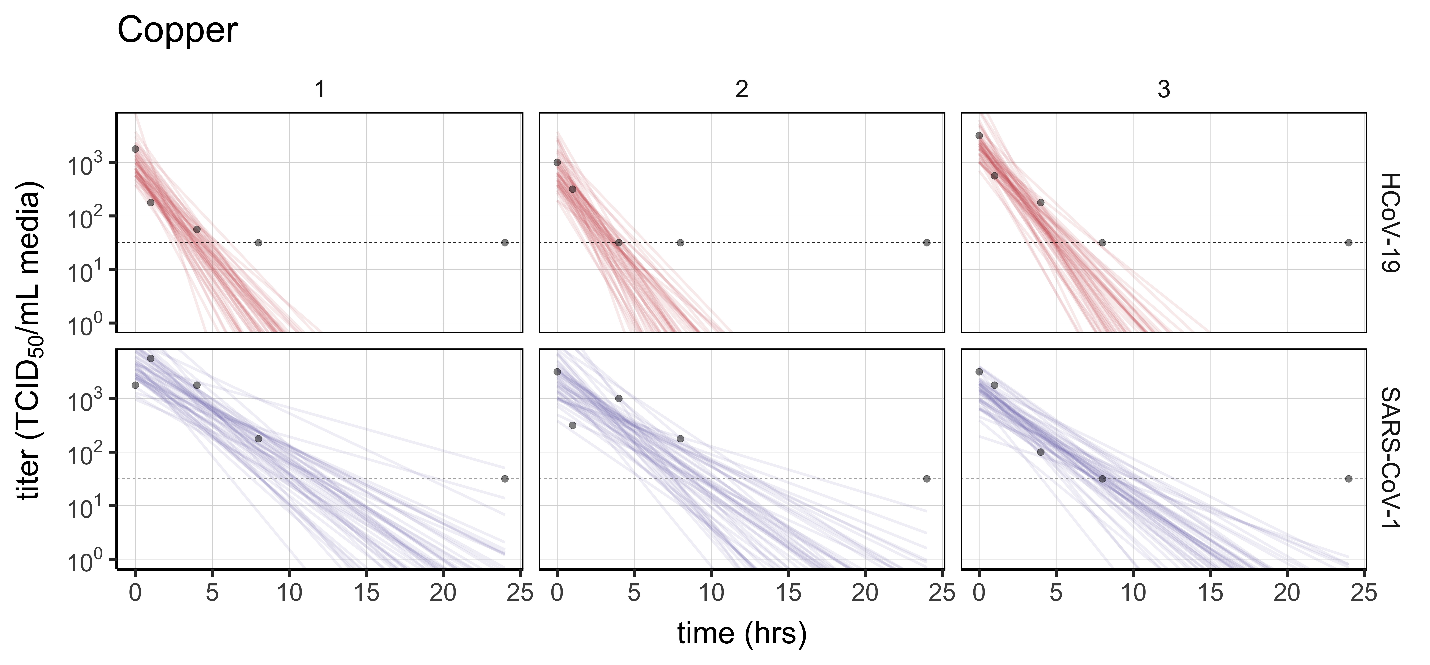


Figure S4. Individual replicate fits for copper. Columns show replicates, rows show virus (HCoV-19 above, SARS-CoV-1 below). Lines are 50 random draws per panel from the posterior distribution of fitted lines, to show level of uncertainty. Fits are substantially poorer for SARS-CoV-1 than for HCoV-19, and data do not follow a linear downward trend over time, suggesting that the difference in observed decay rates should be interpreted with caution.


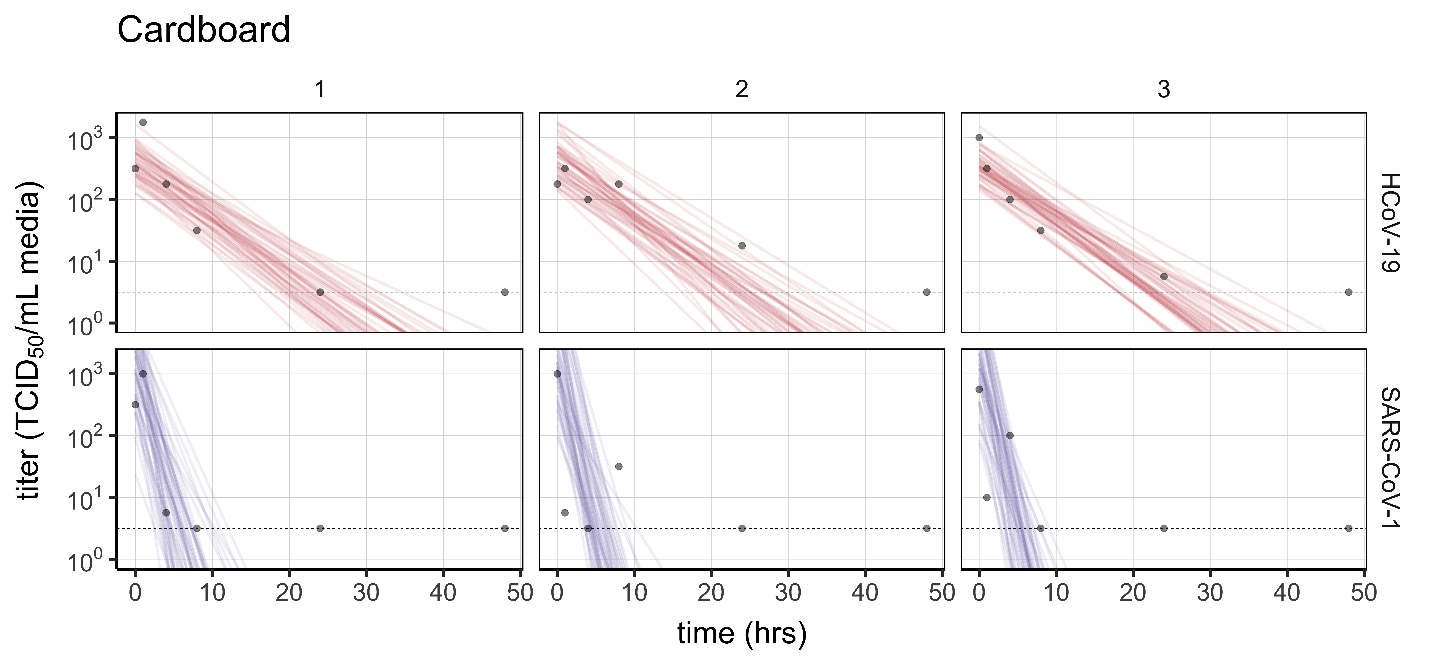


Figure S5. Individual replicate fits for cardboard. Columns show replicates, rows show virus (HCoV-19 above, SARS-CoV-1 below). Lines are 50 random draws per panel from the posterior distribution of fitted lines, to show level of uncertainty. Fits are substantially poorer for SARS-CoV-1 than for HCoV-19, and data do not follow a linear downward trend over time, suggesting that the difference in observed decay rates should be interpreted with caution.

**Supplemental references**

Fischer, R.J., Bushmaker, T., Judson, S., Munster, V.J., 2016. Comparison of the Aerosol Stability of 2 Strains of Zaire ebolavirus From the 1976 and 2013 Outbreaks. J. Infect. Dis. 214, 290–293.

Gelman, A., Carlin, J.B., Stern, H.S., Dunson, D.B., Vehtari, A., Rubin, D.B., 2013. Bayesian Data Analysis, Third Edition. CRC Press.

Holshue, M.L., DeBolt, C., Lindquist, S., Lofy, K.H., Wiesman, J., Bruce, H., Spitters, C., Ericson, K., Wilkerson, S., Tural, A., Diaz, G., Cohn, A., Fox, L., Patel, A., Gerber, S.I., Kim, L., Tong, S., Lu, X., Lindstrom, S., Pallansch, M.A., Weldon, W.C., Biggs, H.M., Uyeki, T.M., Pillai, S.K., 2020. First Case of 2019 Novel Coronavirus in the United States. N. Engl. J. Med. 382, 929–936.

Marra, M.A., Jones, S.J.M., Astell, C.R., Holt, R.A., Brooks-Wilson, A., Butterfield, Y.S.N., Khattra, J., Asano, J.K., Barber, S.A., Chan, S.Y., Cloutier, A., Coughlin, S.M., Freeman, D., Girn, N., Griffith, O.L., Leach, S.R., Mayo, M., McDonald, H., Montgomery, S.B., Pandoh, P.K., Petrescu, A.S., Robertson, A.G., Schein, J.E., Siddiqui, A., Smailus, D.E., Stott, J.M., Yang, G.S., Plummer, F., Andonov, A., Artsob, H., Bastien, N., Bernard, K., Booth, T.F., Bowness, D., Czub, M., Drebot, M., Fernando, L., Flick, R., Garbutt, M., Gray, M., Grolla, A., Jones, S., Feldmann, H., Meyers, A., Kabani, A., Li, Y., Normand, S., Stroher, U., Tipples, G.A., Tyler, S., Vogrig, R., Ward, D., Watson, B., Brunham, R.C., Krajden, M., Petric, M., Skowronski, D.M., Upton, C., Roper, R.L., 2003. The Genome sequence of the SARS-associated coronavirus. Science 300, 1399–1404.

van Doremalen, N., Bushmaker, T., Munster, V., 2013. Stability of Middle East respiratory syndrome coronavirus (MERS-CoV) under different environmental conditions. Eurosurveillance 18, 20590.

Zou, L., Ruan, F., Huang, M., Liang, L., Huang, H., Hong, Z., Yu, J., Kang, M., Song, Y., Xia, J., Guo, Q., Song, T., He, J., Yen, H.-L., Peiris, M., Wu, J., 2020. SARS-CoV-2 Viral Load in Upper Respiratory Specimens of Infected Patients. N. Engl. J. Med. In press.

**Code and data availability**

Code and data to reproduce the Bayesian estimation results and produce corresponding figures are archived online at OSF: <insert link> and available on Github: <insert link>

**Acknowledgements**

We would like to thank Kwe Claude Yinde and Michael Letko for experimental assistance. This research was supported by the Intramural Research Program of the National Institute of Allergy and Infectious Diseases (NIAID), National Institutes of Health (NIH). JOL-S and AG were supported by the Defense Advanced Research Projects Agency DARPA PREEMPT # D18AC00031, and JOL-S was supported by the U.S. National Science Foundation (DEB-1557022) and the Strategic Environmental Research and Development Program (SERDP, RC‐2635) of the U.S. Department of Defense. The findings and conclusions in this report are those of the author(s) and do not necessarily represent the official position of the Centers for Disease Control and Prevention. Names of specific vendors, manufacturers, or products are included for public health and informational purposes; inclusion does not imply endorsement of the vendors, manufacturers, or products by the Centers for Disease Control and Prevention or the US Department of Health and Human Services.
