# Supplementary material for: Aerosol and surface stability of HCoV-19 (SARS-CoV-2) compared to SARS-CoV-1
Source: medRxiv. 2020 Mar 13:2020.03.09.20033217. Originally published 2020 Mar 13. Preprint. [Version 2] doi: 10.1101/2020.03.09.20033217 (PMC7217062; doi:10.1101/2020.03.09.20033217)
Supplement: Supplement 2020 [file 73380-2020.03.09.20033217-1.docx]

**Supplemental Appendix**

# Aerosol and surface stability of SARS-2 (HCoV-19) compared to SARS-CoV

Neeltje van Doremalen^1*^, Trenton Bushmaker^1*^, Dylan H. Morris^2*^, Myndi G. Holbrook^1^, Amandine Gamble^3^, Brandi N. Williamson^1^, Natalie J. Thornburg^4^, Susan I. Gerber^4^, James O. Lloyd-Smith^3,5^, Emmie de Wit^1^, Vincent J. Munster^1^

1. Laboratory of Virology, Division of Intramural Research, National Institute of Allergy and Infectious Diseases, National Institutes of Health, Hamilton, MT, USA
2. Dept. of Ecology and Evolutionary Biology, Princeton University, Princeton, NJ, USA
3. Dept. of Ecology and Evolutionary Biology, University of California, Los Angeles, Los Angeles, CA, USA
4. Division of Viral Diseases, National Center for Immunization and Respiratory Diseases, Centers for Disease Control and Prevention, Atlanta, GA, USA.
5. Fogarty International Center, National Institutes of Health, Bethesda, MD, USA

^*^ These authors contributed equally to this article

# Supplemental methods

Bayesian regression model description

Here we describe in detail the Bayesian regression model that we used to estimate exponential decay rates and half-lives.

In the model notation that follows, the symbol ~ denotes that a random variable is distributed according to the given distribution. Normal distributions are parametrized as Normal(mean, standard deviation). Positive-constrained normal distributions (“Half-Normal”) are parametrized as Half-Normal(mode, standard deviation). We use <Distribution Name>CDF(x, parameters) to denote the cumulative distribution function of a probability distribution, so for example NormalCDF(5, 0, 1) is the value of the Normal(0, 1) cumulative distribution function at 5.

Our data consist of 10 experimental conditions: 2 viruses (HCoV-19 and SARS-CoV) by 5 environmental conditions (aerosols, polypropylene, stainless steel copper and cardboard). Each has three replicates, and multiple time-points for each replicate. We analyze the two viruses separately. For each, we denote by y_ijk_ the measured log_10_ titer in experimental condition *i* during replicate *j* at time-point *k*. To construct our likelihood function, we need to know the probability of observing a given log_10_ titer measurement y_ijk_ given values of the parameters.

Because our titer data are estimated and recorded in increments of 1/n_wells_ log_10_TCID_50_/mL, where n_wells_ is the number of wells used for endpoint titration, our log_10_ titer values are interval-censored – only known to within a range of width 1/n_wells_. In addition, there is a degree of measurement noise in the titration process itself.

To model this, we assume that in each experimental condition *i*, there is a true underlying log_10_ titer x_ijk_ that is measured with some amount of experimental noise or error ε_ijk_ and then observed as an interval-censored value y_ijk_ ≈ x_ijk_ + ε_ijk_. We model the measurement errors ε_ijk_ as Normally distributed with a standard deviation σ_i_ that is shared by all samples in the given experimental condition; this reflects the fact that some experimental setups may be more or less noisy than others.

ε_ijk_ ~ Normal(0, σ_i_)

We model the probability of observing an interval-censored log_10_ titer value y_ijk_ given a true underlying log_10_ titer x_ijk_ and a measurement error standard deviation σ_i_ as:

P(y_ijk_ | x_ijk_, σ_i_ ) = NormalCDF(y_ijk_, x_ijk_, σ_i_) – NormalCDF(y_ijk_ – 1/n_wells_, x_ijk_, σ_i_)

This reflects the probability given a true value x_ijk_ plus the measurement error x_ijk_ + ε_ijk_ falls between y_ijk_ – 1/n_wells_ and y_ijk_. Due to the log_10_ titer imputation technique used, a titer in that range is most likely to be rounded up and reported as y_ijk_.

The detection limit of our experiment is 0.5 log_10_ TCID_50_/mL. The probability of observing an undetectable measured log_10_ titer value y_ijk_ given a true log_10_ titer value x_ijk_ is given by:

P(y_ijk_ ≤ 0.5 | x_ijk_, σ_i_) = NormalCDF(0.5, x_ijk_, σ_i_)

We then model each replicate *j* for experimental condition *i* as starting with some true initial log_10_ titer x_ij_(0) = x_ij0_. We assume that viruses in experimental condition *i* decay exponentially at a rate λ_i_ over time *t*. It follows that

x_ij_(t) = x_ij0_ – λ_i_t

where t_k_ is the k^th^ measured time-point.

Model prior distributions

We place a weakly informative Normal prior distribution on the initial log_10_ titers x_ij0_ to rule out implausibly large or small values (e.g. in this case undetectable log_10_ titers or log_10_ titers much higher than the deposited concentration), while allowing the data to determine estimates within plausible ranges:

x_ij0_ ~ Normal(4.5, 2.5)

We likewise placed a weakly informative Half-Normal prior on the exponential decay rates λ_i_:

λ_i_ ~ Half-Normal(0.5, 4)

We placed a weakly informative Half-Normal prior on the standard deviations of the experimental error distributions σ_i_:

σ_i_ ~ Half-Normal(0, 2)

Markov Chain Monte Carlo Methods

We drew posterior samples using Stan, which implements a No-U-Turn Sampler (a form of Markov Chain Monte Carlo). We ran four replicate chains from random initial conditions for 2000 iterations, with the first 1000 iterations as a warmup/adaptation period. We saved the final 1000 iterations from each chain, giving us a total of 4000 posterior samples. We assessed convergence by inspecting trace plots and examining *R̂* and effective sample size (n_eff_) statistics (*R̂* for all parameters ≤ 1.003, n_eff_ for all parameters ≥28% of total samples).

# Supplemental Figures

Figures S1–S5 show Bayesian fits to individual replicate virus decay data for each virus. Replicates are shown in panel columns, viruses in panel rows. Lines are 50 random draws per panel from the posterior distribution of fitted lines, to show level of uncertainty. Time axis on same scale across all figures, shown out to the latest time taken to reach an undetectable titer (96 hours, for all replicates of both viruses on plastic).


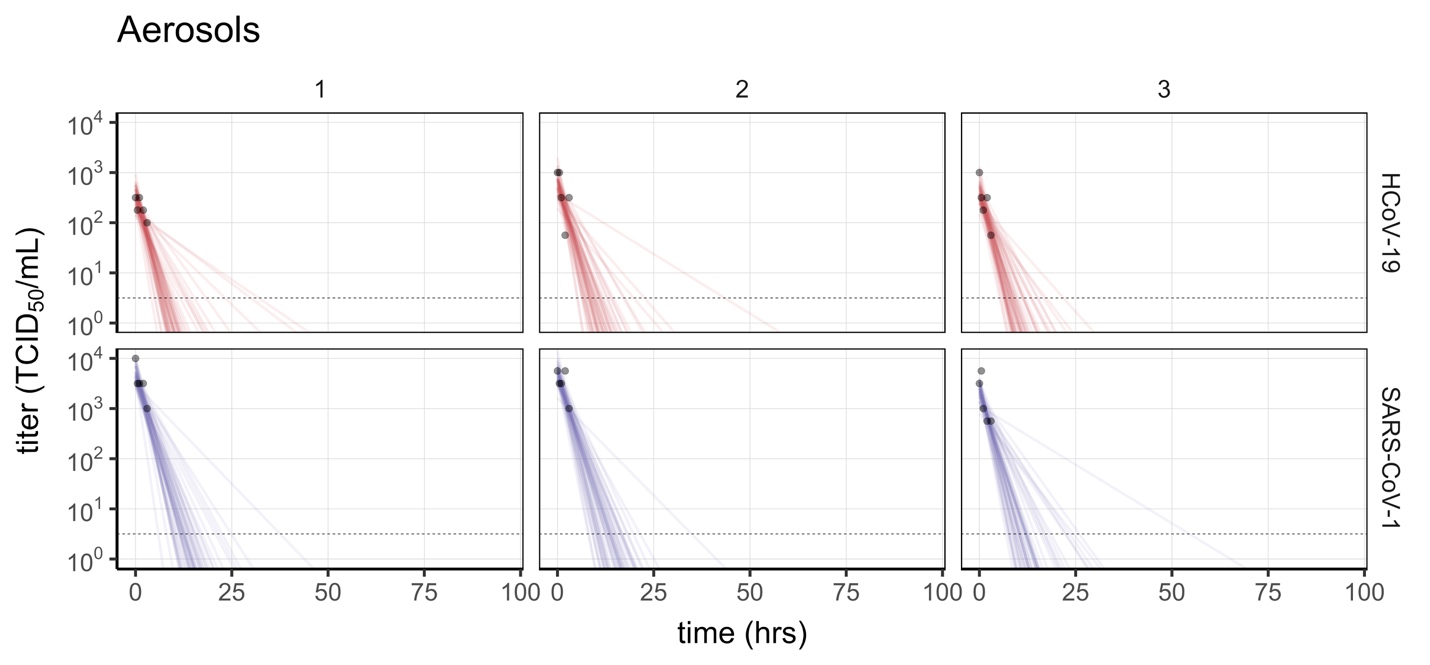


Figure S1. Individual replicate fits for aerosols. Columns show replicates, rows show virus (HCoV-19 above, SARS-CoV-1 below). Lines are 50 random draws per panel from the posterior distribution of fitted lines, to show level of uncertainty.


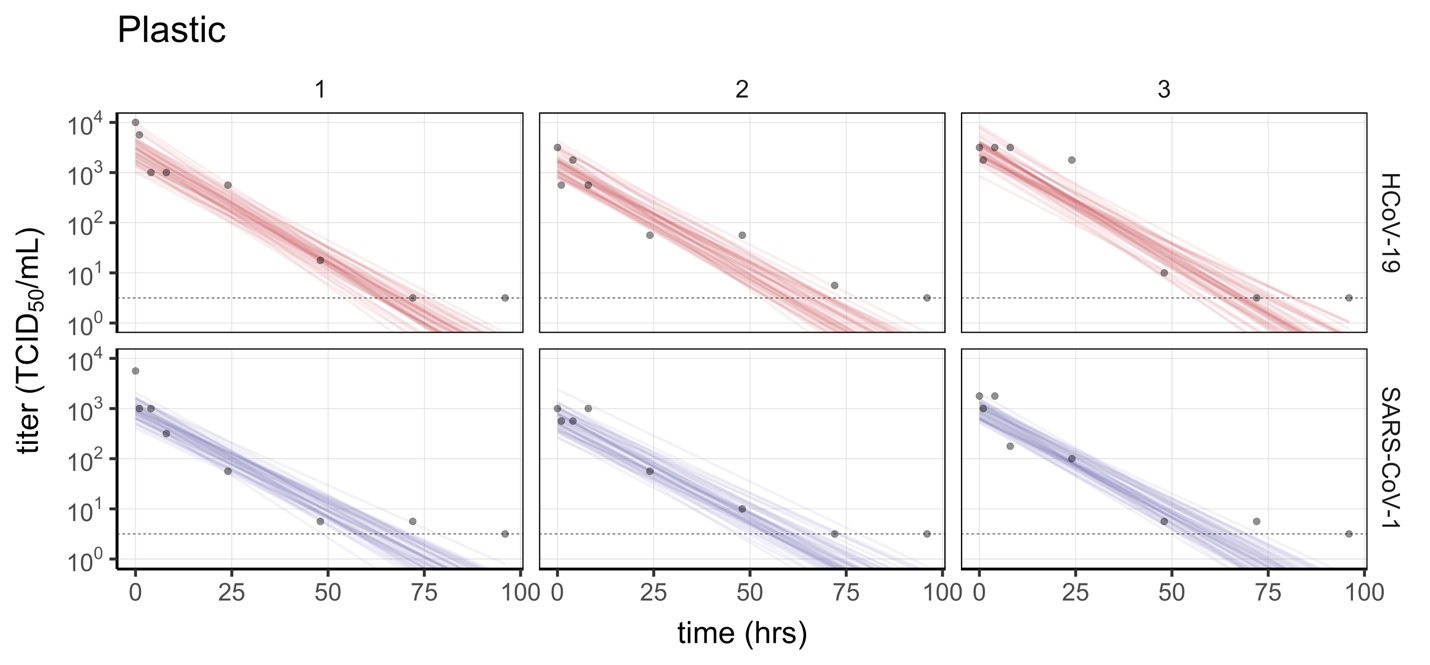


Figure S2. Individual replicate fits for plastic (polypropylene). Columns show replicates, rows show virus (HCoV-19 above, SARS-CoV-1 below). Lines are 50 random draws per panel from the posterior distribution of fitted lines, to show level of uncertainty.


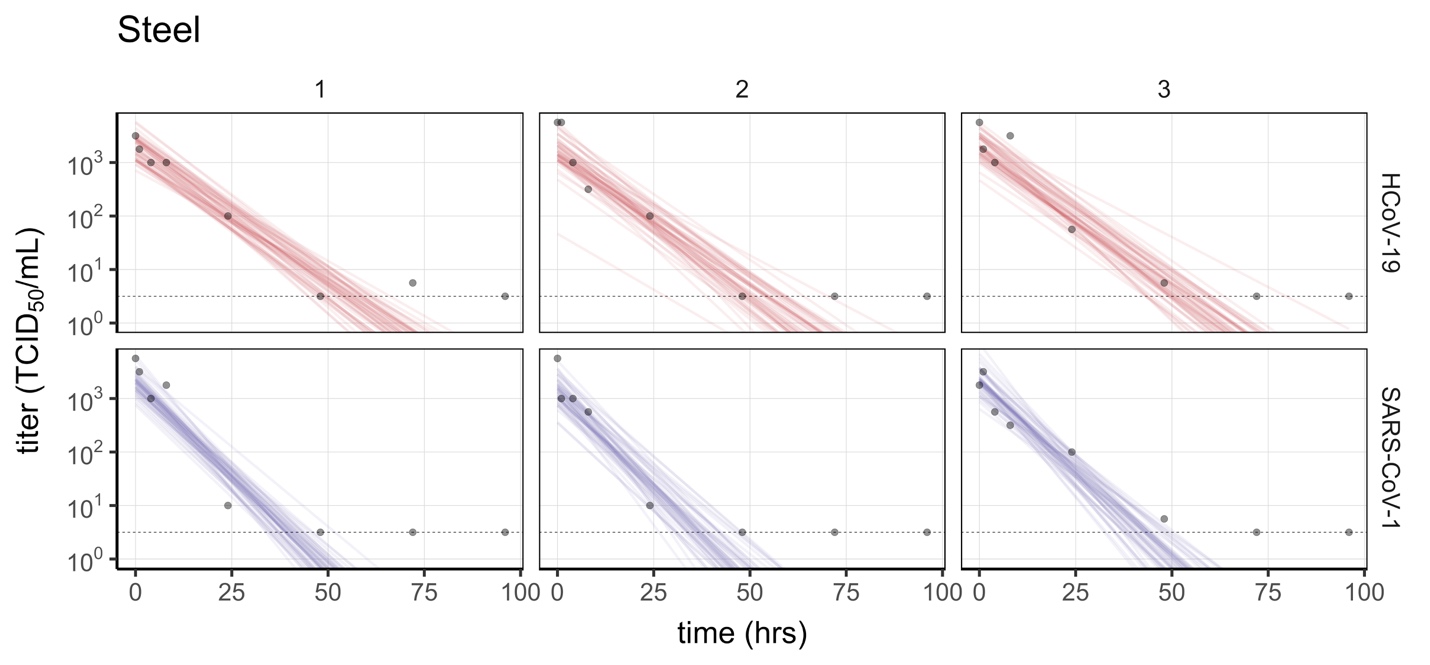


Figure S3. Individual replicate fits for steel. Columns show replicates, rows show virus (HCoV-19 above, SARS-CoV-1 below). Lines are 50 random draws per panel from the posterior distribution of fitted lines, to show level of uncertainty.


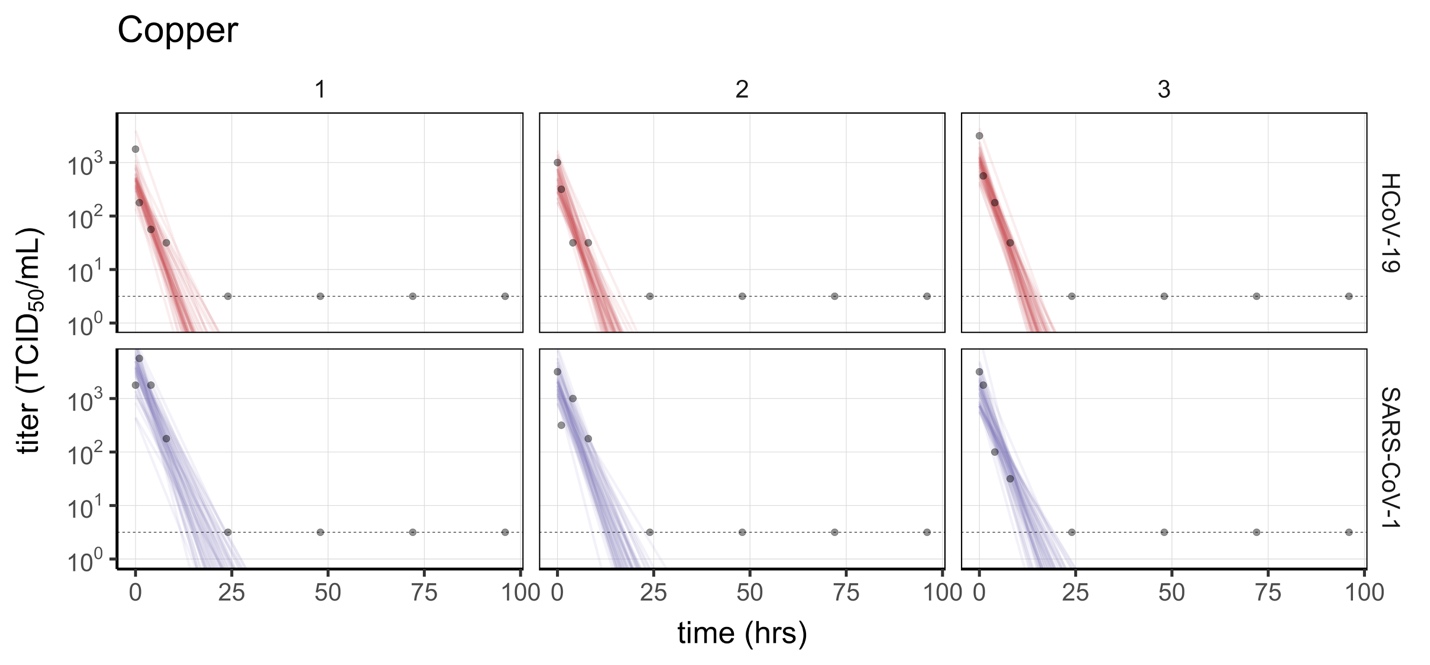


Figure S4. Individual replicate fits for copper. Columns show replicates, rows show virus (HCoV-19 above, SARS-CoV-1 below). Lines are 50 random draws per panel from the posterior distribution of fitted lines, to show level of uncertainty.


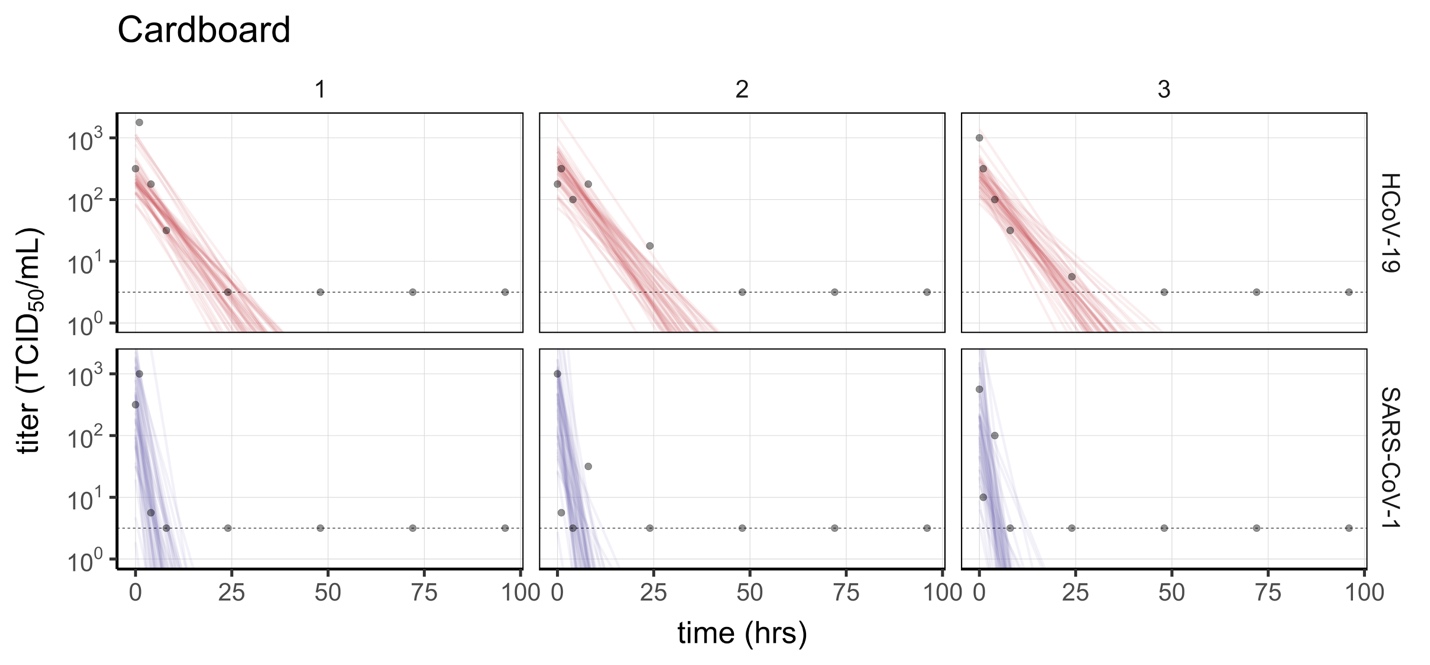


Figure S5. Individual replicate fits for cardboard. Columns show replicates, rows show virus (HCoV-19 above, SARS-CoV-1 below). Lines are 50 random draws per panel from the posterior distribution of fitted lines, to show level of uncertainty. Fits are substantially poorer for SARS than for HCoV-19, and data do not follow a linear downward trend over time, suggesting that the difference in observed decay rates should be interpreted with caution.
